# Unraveling fitness landscapes in plant-associated Bacteroidota

**DOI:** 10.1093/ismeco/ycag186

**Published:** 2026-07-01

**Authors:** Marta Torres, Vincent Lombard, Nicolas Terrapon, Adam M Deutschbauer

**Affiliations:** Environmental Genomics and Systems Biology Division, Lawrence Berkeley National Laboratory, Berkeley, CA 94720, United States; Aix Marseille University, CNRS, UMR7257, AFMB, Marseille 13288, France; INRAE, USC1408 AFMB, Marseille 13288, France; Aix Marseille University, CNRS, UMR7257, AFMB, Marseille 13288, France; INRAE, USC1408 AFMB, Marseille 13288, France; Environmental Genomics and Systems Biology Division, Lawrence Berkeley National Laboratory, Berkeley, CA 94720, United States; Department of Plant and Microbial Biology, University of California, Berkeley, CA 94720, United States

**Keywords:** Bacteroidetes, *Mucilaginibacter*, rhizosphere, *Brachypodium distachyon*, randomly barcoded transposon sequencing (RB-TnSeq), polysaccharide utilization loci (PULs), carbohydrate-active enzymes (CAZymes)

## Abstract

Members of the bacterial phylum Bacteroidota inhabit diverse ecosystems, yet environmental species remain poorly understood compared to clinically relevant ones. Although Bacteroidota are dominant in crop-associated microbiomes, the mechanisms enabling them to thrive in plant and soil environments remain unclear. A key obstacle in understanding gene fitness in Bacteroidota is the lack of genome-wide functional studies, largely due to their inherent resistance to antibiotics, which hinders genetic manipulation. Here, we applied randomly barcoded transposon mutagenesis sequencing (RB-TnSeq) to measure gene fitness in a plant-associated Bacteroidota, *Mucilaginibacter yixingensis* YX-36. Our data sheds light on pathways involved in rhizosphere colonization, gliding motility, stress tolerance, and carbon metabolism. Notably, we found that phylum-specific genes such as polysaccharide utilization loci and carbohydrate-active enzymes are needed for fitness in the plant niche. Overall, this work advances our understanding of gene functions in environmental Bacteroidota species and provides a foundation for future research on their roles in plant-microbe interactions.

## Introduction

Members of the phylum Bacteroidota live in a wide range of environments, including water, soil, plants, and animals. In animal hosts, they colonize skin, the oral cavity, and especially the gastrointestinal tract, where they make up a major fraction of the bacteriome and contribute to nutrient supply, immune regulation, and disease protection [1, 2]. Although well-studied as dominant gut microbiota members [3], their ecological roles in other systems, such as plant microbiomes, are still relatively underexplored [4].

Plants represent a major source of organic carbon in the environment, largely because they are rich in glycans. These organics range from simple sugars to complex, recalcitrant polysaccharides that persist in soil [5]. Plant glycans make up a large fraction of plant biomass and root mucilage and have been well characterized in models such as *Brachypodium distachyon* [6, 7], whose cell wall is composed of arabinoxylans, cellulose, and β-glucans [8]. While fungi are often considered the main degraders of plant polysaccharides, soil bacteria including Bacteroidota are increasingly recognized as key contributors [9, 10].

Bacteroidota are found in plant-associated environments including agricultural soils [11] and forest ecosystems [12]. They typically represent a large fraction of the root microbiome [13, 14] and are also found in the phyllosphere of gymnosperms [15, 16]. In some angiosperms, they are the most enriched phylum in roots and rhizosphere compared with bulk soil [17]. They also appear more abundant in wild crops than in domesticated varieties [18–20]. Because Bacteroidota respond strongly to agricultural practices, they have been proposed as indicators of soil health [21, 22]. In addition, they may also function as microbial inoculants, replacing traditional fertilizers, as some species have demonstrated plant growth-promoting and/or stress-alleviating effects [23–25].

The broad distribution of Bacteroidota reflects their strong adaptability. Environmental Bacteroidota typically have larger genomes, consistent with metabolic versatility and broad catabolic capacity [1, 26, 27]. They are recognized specialists in degrading high-molecular-weight organic matter, particularly polysaccharides and proteins [28]. Their success depends on large repertoires of carbohydrate-active enzymes (CAZymes) [29] that target diverse plant glycans (e.g. pectins, hemicelluloses like xylans) [5, 28]. Interestingly, terrestrial Bacteroidota show especially high CAZyme abundance and diversity [4], supporting their adaptation to plant carbohydrate-rich niches [10, 30].

Polysaccharide degradation in Bacteroidota is coordinated through polysaccharide utilization loci (PULs), which group CAZymes, transporters, and regulators into substrate-responsive modules [28]. Their capabilities are further amplified by the Type IX Secretion System (T9SS), which exports CAZymes to the cell surface and is closely linked to the ability to glide rapidly across surfaces, thereby enabling active nutrient hunting [5, 31]. This strategy allows them to sequester oligosaccharides and degrade them in the periplasm, reducing resource sharing with competitors [32]. They also influence soil microbial communities by triggering cross-feeding networks [33, 34], although resource release can reduce their own fitness [35].

Understanding the mechanisms that allow Bacteroidota to colonize plant roots could create opportunities for enhancing crop production through microbiome engineering [9]. To investigate the genetic determinants of rhizosphere colonization in environmental Bacteroidota, we attempted randomly barcoded transposon sequencing (RB-TnSeq) [36] in representatives of various soil and plant-related genera, including *Flavitalea, Flavisolibacter, Niastella, Mucilaginibacter, Chitinophaga, Runella, Sediminibacterium, Spirosoma*, and *Dyadobacter*. Due to widespread antibiotic resistance in these strains, success was achieved only for *Mucilaginibacter yixingensis* YX-36, originally isolated from a vegetable field [37]. *Mucilaginibacter* species are abundant in agricultural soils [38], promote plant growth [25, 39, 40], and are frequently included in synthetic communities (SynComs) designed to be used in *B. distachyon* [41, 42]. Yet their genetic traits for rhizosphere colonization remain unknown. Our results show that gliding motility and degradation of complex carbon sources are key determinants of *M. yixingensis* root colonization. The resources developed here will facilitate future functional studies in environmental Bacteroidota.

## Material and methods

### Bacterial strains and culture conditions

*Escherichia coli* strains were cultured in LB at 37°C. Bacteroidota strains including members of an established switchgrass synthetic community [43] and strains from public repositories (Table 1) were cultured in R2A at 30°C. If appropriate, 180 rpm rotary shaking was used. Antibiotic susceptibility was conducted in R2A with tetracycline (Tc), chloramphenicol (Cm), streptomycin (Str), kanamycin (Km), erythromycin (Erm), or gentamicin (Gm) at a final concentration of 50 or 100 μg/ml. When needed, diaminopimelic acid (DAP) was added to a final concentration of 300 μM, and phenylalanine-arginine-β-naphthylamide (PAβN) was used at 25 μg/ml.

**Table 1:** Bacteroidota environmental strains used in this study.

Strain	Isolated from	Origin
**Family Chitinophagaceae**		
*Chitinophaga* sp. OAE627	Soil, field with switchgrass	Shared by Kateryna Zhalnina (Lawrence Berkeley National Laboratory)
*Chitinophaga* sp. OAE851	Soil, field with switchgrass	Shared by Kateryna Zhalnina (Lawrence Berkeley National Laboratory)
*Chitinophaga* sp. OAE863	Soil, field with switchgrass	Shared by Kateryna Zhalnina (Lawrence Berkeley National Laboratory)
*Chitinophaga* sp. OAE865	Soil, field with switchgrass	10.1128/msystems.00951-22
*Chitinophaga* sp. OAE868	Soil, field with switchgrass	Shared by Kateryna Zhalnina (Lawrence Berkeley National Laboratory)
*Chitinophaga* sp. OAE870	Soil, field with switchgrass	Shared by Kateryna Zhalnina (Lawrence Berkeley National Laboratory)
*Chitinophaga* sp. OAE911	Soil, field with switchgrass	Shared by Kateryna Zhalnina (Lawrence Berkeley National Laboratory)
*Chitinophaga* sp. OAS766	Soil, field with switchgrass	Shared by Kateryna Zhalnina (Lawrence Berkeley National Laboratory)
*Chitinophaga* sp. OAS790	Soil, field with switchgrass	Shared by Kateryna Zhalnina (Lawrence Berkeley National Laboratory)
*Chitinophaga pinensis* UQM2034	Pine tree litter	10.1099/00207713-31-3-285; obtained from DSMZ collection
*Flavisolibacter ginsenosidimutans* Gsoil636	Soil of ginseng field	10.1099/ijsem.0.000660; obtained from DSMZ collection
*Flavitalea* sp. OAE831	Soil, field with switchgrass	Shared by Kateryna Zhalnina (Lawrence Berkeley National Laboratory)
*Sediminibacterium ginsengisoli* DCY13	Soil of ginseng field	10.1099/ijs.0.038554-0; obtained from DSMZ collection
**Family Cytophagaceae**		
*Dyadobacter beijingensis* A54	Rhizospheric soil of turf grass	10.1099/ijs.0.64754-0; obtained from DSMZ collection
*Niastella koreensis* GR20-10	Soil of ginseng field	10.1099/ijs.0.64242-0; obtained from DSMZ collection
*Niastella* sp. OAE872	Soil, field with switchgrass	Shared by Kateryna Zhalnina (Lawrence Berkeley National Laboratory)
*Niastella* sp. OAS944	Soil, field with switchgrass	10.1128/msystems.00951-22
*Niastella yeongjuensis* GR20-13	Soil of ginseng field	10.1099/ijs.0.64242-0; obtained from DSMZ collection
*Runella zeae* NS12	Maize plant stem	10.1099/00207713-52-6-2061; obtained from DSMZ collection
**Family Sphingobacteriaceae**		
*Mucilaginibacter gracilis* TPT18	moss peat bog	10.1099/ijs.0.65100-0; obtained from DSMZ collection
*Mucilaginibacter* sp. OAE612	soil, field with switchgrass	10.1128/msystems.00951-22
*Mucilaginibacter polytrichastri* RG4–7	moss	10.1099/ijs.0.055335-0; obtained from DSMZ collection
*Mucilaginibacter yixingensis* YX-36	vegetable soil	10.1099/ijsem.0.000941; obtained from DSZM collection
**Family Spirosomataceae**		
*Spirosoma endophyticum* EX36	xylem of willow tree	10.1099/ijs.0.052654-0; obtained from DSMZ collection

### Construction of barcoded mutant libraries in environmental Bacteroidota

#### Identification of an optimal vector for transposon mutagenesis using the magic pool approach

This methodology is a combinatorial strategy for evaluating thousands of uniquely assembled transposon delivery vectors from a collection of DNA parts (transposases, promoters, and selection markers) [44]. It streamlines the process of identifying vectors that have the optimal combination of parts by simultaneously testing the efficacy of all possible vectors in the catalog [45]. Once a vector construction (i.e. the different parts) is identified as the most efficient, it can be reassembled and used to generate a final mutant library. The erythromycin magic pool used in this study (AMD1241) was previously described by our lab [44]. It is a mix of Tn5 (AMD279) and *mariner* (AMD280) magic pools, accounting for >2450 different vector combinations.

Conjugations between the Erm-sensitive Bacteroidota and AMD1241 were conducted in both LB and R2A supplemented with DAP using conjugation disks. After 16 h, the conjugation was scraped off, resuspended in LB or R2A, and plated in selective media with Erm, with and without PAβN. Plates were incubated at 30°C until visible colonies developed. When possible, preliminary libraries were constructed by pooling around 1000 colony forming units and growing them for two population doublings in liquid media. We then performed TnSeq to characterize each mutant library by mapping the insertion location and its associated random DNA barcode as previously described [36], thereby simultaneously assessing the efficacy of the vectors in the magic pool. To map the insertion sites of the transposon mutants, and to link them to their associated DNA barcodes, we used a modified version of our previously described TnSeq protocol [36], using two rounds of PCR to selectively enrich for transposon junctions [46].

#### Construction of a final mutant library with the optimal transposon delivery vector

Once a vector design in the magic pool was identified as the most effective for mutagenesis, the single reassembled vector with the optimal combination of parts was used to construct a final RB-TnSeq [36] transposon mutant library. We only identified an efficient vector for *M. yixingensis* YX-36, and this vector was previously constructed: pTGG43_NN2 [44]. Conjugation between YX-36 and an *E. coli* conjugation donor carrying pTGG43_NN2 was performed as explained above. Plates were incubated at 30°C for 48 h. We then pooled thousands of colonies and grew the library in liquid R2A supplemented with Erm, added glycerol to a final volume of 15%, made multiple 1-ml −80°C freezer stocks (~108 cells/ml) of the final library for subsequent experiments, and collected cell pellets to extract genomic DNA for TnSeq mapping. The final library was called *Mucilaginibacter*_YX36_ML5.

### Fitness assays with *Mucilaginibacter*_YX36_ML5

Plant and *in vitro* conditions (Table S1) were chosen according to traits previously described in *Mucilaginibacter* species and in general, other environmental Bacteroidota. These include colonization of the rhizosphere of *Brachypodium* plants [41, 42], degradation of polysaccharides [10, 47, 48], and tolerance to phosphate [49] and metals [50].

Every time experiments were conducted, one aliquot of the glycerol stocks containing the *Mucilaginibacter*_YX36_ML5 transposon library was thawed and inoculated in 25 ml fresh R2A with 50 μg/ml Erm and grown for approximately 16 h at 30°C and 180-rpm shaking until the culture reached OD600 ~ 1.3. Time0 samples enumerating the relative abundance of each mutant were collected by centrifuging 1 ml aliquots and frozen at −20°C until further DNA purification. The remaining cells were then washed twice with a chemically-defined media without a carbon source (RCH2_defined_noCarbon) [51] prior to inoculation.

#### In vitro fitness assays

Assays were conducted in defined media or rich media depending on the type of experiment, at starting OD_600_ = 0.03. Carbon assays were conducted in RCH2_defined_noCarbon [51] supplemented with different carbon sources (i.e. monosaccharides, disaccharides, polysaccharides). After 24 or 48 h, cultures with an OD higher than the average non-inoculated controls plus two times the standard deviation were centrifuged. Stress assays were conducted in R2A supplemented with compounds at inhibitory concentrations. Temperature assays were conducted in R2A. After 24, 48, 72 h, or 96 h, cultures with an OD lower than that of the no-stress controls were centrifuged. Pellets were kept frozen at −20°C until further DNA purification.

For gliding motility assays, the mutant library was inoculated into the center of 0.5% w/v agar R2A plates, and “outer” samples with motile cells were removed with a sterile razor after 24 h [51]. Cells were harvested from agar by centrifugation, and pellets were kept at −20°C until DNA purification.

#### Plant colonization fitness assays

*Brachypodium distachyon* Bd21-3 seeds were sterilized and germinated as described previously [52]. Seedlings were then transferred to Magenta GA-7 plant culture boxes containing ~150 ml of 0.5× Murashige & Skoog (MS) [53] basal salts (MSP01, Caisson Laboratories, United States) with 0.3% (w/v) agar, where they were grown under conditions previously described [52]. Fifteen days after germination, 1 ml of *Mucilaginibacter*_YX36_ML5 washed cells (OD_600_ = 1) were diluted in 5 ml of 0.5× MS, and the whole volume was inoculated into the plants by injecting directly on the MS agar substrate in 6–7 different spots at ~1 cm around the plant stem. The substrate was stirred with a sterile spatula around the plant to homogenize the bacterial inoculum. Two harvest points were assayed, 7 and 21 days post-inoculation (dpi). Briefly, plants were uprooted, and isolated roots were collected in 50-ml falcons containing 20 ml of R2A and vortexed for 2 minutes at full speed. This sample was referred to as the “rhizosphere.” Roots were then discarded, and the liquid content was transferred to glass tubes. Samples were then incubated (outgrown) for 16 h at 30°C and 180-rpm shaking in R2A. Subsequently, 2-ml aliquots were collected from each sample centrifuged at 3000 *g* for 3 min, and the resulting pellets were stored at −20°C until DNA extraction.

### DNA isolation, library preparation, sequencing, and fitness value calculation

Total genomic DNA from frozen pellets was extracted using the DNeasy Blood & Tissue Kit (Qiagen, Germany). Purified RNA-free DNA was used as a template for DNA barcode PCR amplification under previously described conditions [36]. Strain and gene fitness were calculated using the computation pipeline developed by Wetmore et al. [36]; code available at bitbucket.org/berkeleylab/feba/src/master. Fitness values for each gene were calculated as the log_2_ of the ratio of relative barcode abundance for mutants in that gene following library growth in a given condition (i.e. plant colonization) divided by relative abundance in the Time0 (input) sample. A *t*-like statistic [36] was also determined for every gene in each experiment, which takes into account the consistency of the fitness of all the mutants of that gene to assess if the fitness value is reliably different from 0. To exclude weak colonization fitness values that may lack biological relevance, analyses were restricted to genes with |fitness| ≥ 1 and an associated |t| ≥ 3.

### Data analysis and data availability

Analysis of gene fitness values was done in R [54]. Graphs and heatmaps were plotted in R using the *ggplot2* package [55]. Raw sequencing data is available at https://doi.org/10.6084/m9.figshare.30731249.v1. The majority of the fitness data from this study is publicly available at the Fitness Browser website (https://fit.genomics.lbl.gov) [51] for comparative analysis. Experiments that met or did not meet our thresholds for inclusion in the Fitness Browser are listed in Table S2. The PUL database (PULDB) [56] (www.cazy.org/PULDB/) was used to identify PULs. The CAZy database (www.cazy.org [29]) was used to annotate CAZy domains, with automated annotation for sequences reaching significant coverage and similarity to previously annotated full-length sequences, and manual curation for sequences with low similarity. ​.

## Results and discussion

### Erythromycin and gentamicin are the best candidates for antibiotic selection of environmental Bacteroidota

**Figure 1:**
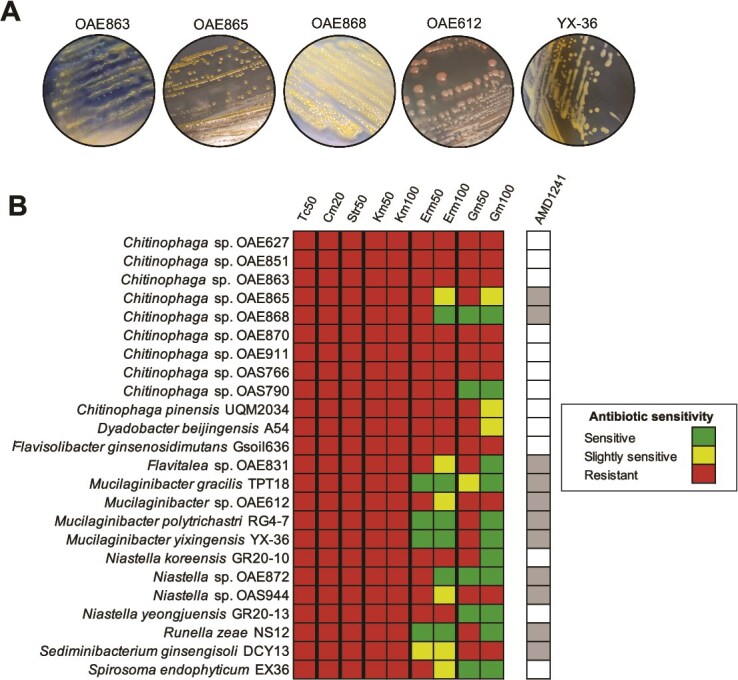
(A) Overview of some of the tested isolates in R2A medium. (B) Sensitivity of environmental Bacteroidota strains to tetracycline (Tc), chloramphenicol (Cm), streptomycin (Str), kanamycin (Km), erythromycin (Erm), and gentamicin (Gm). Erm-sensitive strains in which the combined *mariner* and Tn5 magic pool (AMD1241) was used are highlighted. Details on the listed strains can be found in Table 1.

To determine which antibiotics could be used for selecting mutants in environmental Bacteroidota, we evaluated the sensitivity of 24 strains available in our lab to six antibiotics commonly used in bacterial genetics. We found that the strains tested were naturally resistant to most antibiotics, including Tc, Cm, Str, and Km. Only Erm and Gm were effective on ~50% of the strains (Fig. 1), albeit high concentrations (100 μg/ml) were required for some strains. Given our past success with Erm selection as a marker for making RB-TnSeq libraries, we focused our efforts on the Erm-sensitive strains. We tested the combined *mariner* and Tn5 magic pool [44] with Erm selection on strains *Chitinophaga* sp. OAE865, *Chitinophaga* sp. OAE868, *Niastella* sp. OAE872, *Niastella* sp. OAS944, *Flavitalea* sp. OAE831, *Mucilaginibacter* sp. OAE612, *Mucilaginibacter gracilis* TPT18, *M. yixingensis* YX-36, *Mucilaginibacter polytrichastri* RG4-7, *Runella zeae* NS12, *Spirosoma endophyticum* EX36, and *Sediminibacterium ginsengisoli* DCY13 (Fig. 1). Despite trying different approaches (e.g. different conjugation media, type of transposon delivery vector, selection with different antibiotic concentration, and use of the efflux inhibitor PAβN to reduce antibiotic resistance [57, 58]), we only succeeded in generating transposon mutants in strain YX-36 (Fig. S1).

### Construction of a barcoded mutant library in *M. yixingensis* YX-36

**Figure 2:**
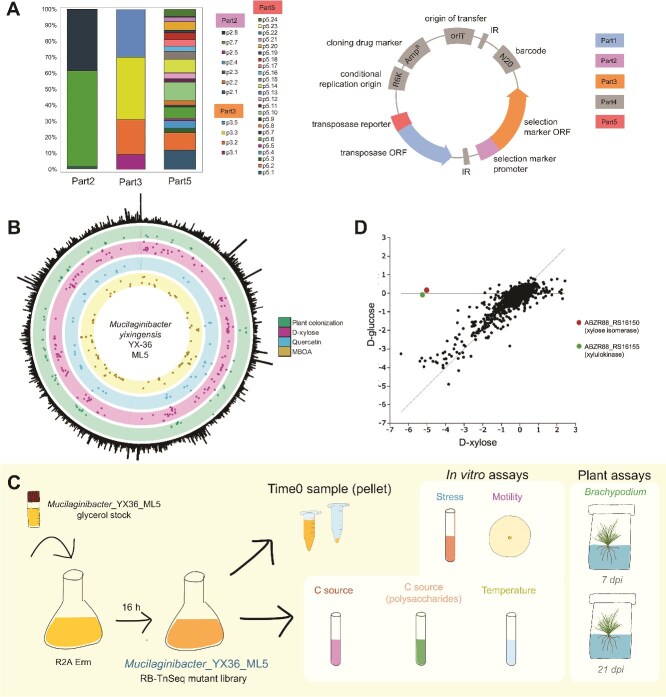
(A) Preference of *Mucilaginibacter yixingensis* YX-36 for different vector parts in the Erm *mariner* magic pool. The fraction of each of part2, part3, and part5 variants in the preliminary mutant library for YX-36 as determined by DNA barcodes identified by TnSeq is shown. DNA part sequences and sources are available from Liu *et al.* [44]. The basic structure of the transposon delivery vector with the different parts indicated in colors is indicated; figure adapted from [44, 45]. (B) Genome-wide map of YX-36 fitness genes. From outside to inside: total reads per gene from TnSeq mapping data, genes involved in plant colonization 7 days post inoculation, during growth in defined minimal media with D-xylose as the carbon source, in stress tolerance to quercetin and 6-methoxy-2(3H)-benzoxazolone (MBOA). (C) Overview of the RB-TnSeq *in vitro* and plant fitness assays conducted with *Mucilaginibacter*_YX36_ML5. Time0 (input) samples are the common control for all experimental conditions. A summary of the conditions tested can be found in Table S1. (D) Correlation between averaged fitness data for D-xylose and D-glucose. ABZR88_RS16155 (coding for xylulokinase) and ABZR88_RS16150 (encoding for xylose isomerase) are shown.

Despite the challenge of constructing mutant libraries in the tested Bacteroidota, we got promising results for *M. yixingensis* YX-36 (NCBI taxonomy ID: 1295612) (Fig. S1). Although a draft genome assembly was already available (NCBI RefSeq assembly GCF_003050755.1), this assembly was incomplete and in multiple contigs. To complete the genome, we generated PacBio reads and assembled them with Flye (NCBI RefSeq assembly GCF_041080815.1) [59]. Using the complete genome, we mapped transposon insertions generated by the e magic pool to identify what genetic 'parts' [44] were more effective for transposon mutagenesis in strain YX-36. The results showed that for part2 (the promoter for the drug resistance CDS) and part3 (the drug resistance CDS), the part2.7 and part3.3 were the most effective (see section 2.2; Fig. 2A). For part5 (promoter for the transposase), the promoters driving the *mariner* transposase gave ~25× more mapped reads than the Tn5 transposase. Several specific part5’s seemed to work more or less similarly including part5.18 (Fig. 2A). Based on these data, we decided to use the single reassembled vector pTGG43_NN2 (in *E. coli* WM3064, strain AMD691) that was previously used to make RB-TnSeq libraries in the Bacteroidota species *Echinicola vietnamensis, Pedobacter* sp. and *Pontibacter actiniarum* [44]. This vector is a combination of some the identified optimal parts for YX-36: part1 (*mariner* transposase), part2.7 (*dnaE* promoter from *Bacteroides thetaiotaomicron*), part3.3 (*ermBP* Erm resistance gene from pHLL24 [60]), part4 (barcoded backbone) and part5 mar_p5.18 (Dxs promoter from *Belliella baltica*).

The final RB-TnSeq *mariner* library was termed *Mucilaginibacter*_YX36_ML5. TnSeq mapping of transposon insertion sites resulted in a pool of 61 393 mutant strains with mapped insertions and unique barcodes at 56102 different locations distributed across the genome (Fig. 2B). We mapped at least one central mutation in 3799 genes of the 4390 predicted protein-coding genes (86.5% of the YX-36 genome). Using this RB-TnSeq library, we collected fitness data for plant colonization, carbon catabolism, motility, temperature, and stress tolerance (Fig. 2C, Table S2) for 3566 genes. The remaining genes lack gene fitness data because they are represented by few or no mutants in the library (many of which are likely essential) or because mutants in these genes had low read counts in the Time0 samples (Table S3).

For each experiment that we conducted with *Mucilaginibacter*_YX36_ML5, gene fitness values were calculated (see section 2.5). A negative fitness (fitness ≤1) means that the mutants in that gene were depleted in that given condition; these are called 'important' genes. A positive fitness (fitness ≥1) means that the mutants in that gene are enriched in that condition relative to the typical mutant in the library; these are called 'detrimental' genes.

### Validation of the *Mucilaginibacter*_YX36_ML5 mutant library

After generation of *Mucilaginibacter*_YX36_ML5, we used it with single monosaccharide compounds with the aim to validate the constructed library. The results obtained (Table S2) show that our mutant library is accurate, which validates it as a promising tool for gene function studies in environment-related *Mucilaginibacter* species. Specifically, the adjacent genes ABZR88_RS16155 (xylulokinase) and ABZR88_RS16150 (xylose isomerase) are important for metabolism of D-xylose (Fig. 2D), the main residue in plant xylans [5], but not of other carbon sources we profiled including D-glucose. The role of these genes in xylose metabolism is well characterized in model organisms [61]. Another example of such validation is ABZR88_RS03755 (carbohydrate kinase/fructokinase, the limiting enzyme in fructose metabolism), which is only needed for growth in our experiments with D-fructose as the sole carbon source (Table S2).

### Identification of genes contributing to fitness in strain YX-36 across diverse *in vitro* conditions

In order to gain insight into different lifestyles and abilities related to environmental Bacteroidota [25, 47], we tested the YX-36 mutant library across conditions such as metabolism of carbon compounds, gliding motility, and stress tolerance to different compounds.

#### Metabolism of carbon sources

Bacteroidota are mainly able to orchestrate the degradation of complex glycans by organizing the key players into PULs, which allow high-level production of specific CAZymes only when the corresponding substrate is abundant [5, 28]. PULs are sets of co-localized genes that usually encode a SusCD pair: a TonB-dependent transporter (SusC) that works with a glycan-capturing lipoprotein (SusD). In Bacteroidota glycan-degrading systems, secreted CAZymes partially depolymerize the polysaccharide into oligosaccharides, which are imported into the periplasm by SusCD transporters, and subsequently broken down there by additional sugar-cleaving enzymes, keeping the products away from competing organisms [28].

One powerful tool to identify PULs in Bacteroidota strains is the PULDB [56], which displays predicted PULs and CAZyme clusters (devoid of SusCD). Among the >40 *Mucilaginibacter* genomes in PULDB, the number of predicted PULs ranges from 13 in *M. ginkgonis* HMF7856 to almost a hundred in *M. paludis* DSM 18603. In comparison, 45 PULs are predicted in *M. yixingensis* YX-36 (Fig. S2, Table S4). When the specificities of CAZyme (sub)families gather in a PUL match known glycan structure, it allows to predict the PUL substrates and likely growth ability of the species. For example, in YX-36*,* PUL22, PUL32, and PUL44 likely target xylan, ⍺-1,4/6-glucans (e.g. starch, pullulan), and β-1,3-glucans (e.g. plant mixed-β-1,3/4-glucans or fungal β-1,3-glucans), respectively. Additional PULs are predicted to contain CAZymes that target complex and intertwined pectin polysaccharides.

**Figure 3:**
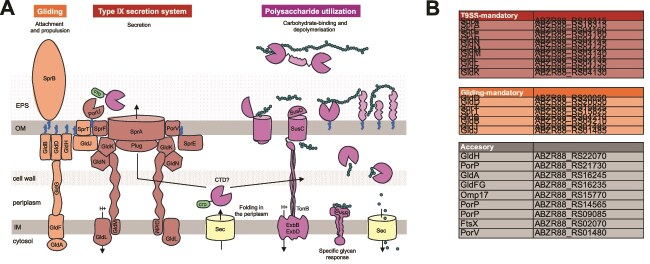
(A) Overview of PULs and gliding motility mediated by the type IX secretion system (T9SS). EPS, exopolysaccharide; IM, inner membrane; OM, outer membrane. Blue wavy lines on proteins symbolize lipid anchors for lipoproteins. Figure modified from Larsbrink and McKee [5]. (B) T9SS and gliding mandatory components in *Mucilaginibacter yixingensis* YX-36, as assessed with the T9GPred tool [70].

CAZy annotation of the YX-36 genome assembly shows a high number of enzymes belonging to different families (Table S4). More precisely, we found 95 glycosyltransferases (GT), which form glycosidic bonds; 35 carbohydrate esterases (CE), which hydrolyze carbohydrate esters; 16 carbohydrate-binding modules (CBM), which adhere to carbohydrates; 202 glycoside hydrolases (GH), which hydrolyze and/or rearrange glycosidic bonds; and 32 polysaccharide lyases (PL), which perform non-hydrolytic cleavage of glycosidic bonds. The number of PLs and CEs in *M. yixingensis* is the highest among the *Mucilaginibacter* species currently annotated (Figs S3 and S4). Our computational analysis agrees with previous findings on high numbers of CAZymes in the genomes of *Mucilaginibacter* species, especially GHs [10]. While CAZymes can also be secreted by the phylum-specific T9SS (Fig. 3A) [5], in strain YX-36 we only found two PLs that display a C-terminal domain (CTD) indicating their secretion by the T9SS.

To represent the diversity of compounds degraded by environmental Bacteroidota, we selected different polysaccharides, disaccharides, and monosaccharides. For plant-related polysaccharides we used ⍺-1,4-glucans (e.g. starch, polygalacturonic acid) and β-1,4-glucans (e.g. carboxymethylcellulose). As disaccharides, we used derivatives of the above polysaccharides; including D-cellobiose (a subproduct of carboxymethylcellulose) or D-maltose (produced after the hydrolysis of starch). As monosaccharides we used basic units of the above including D-glucose, L-arabinose, and L-rhamnose. From the 14 carbon sources that we profiled, we identified genes specifically important for the catabolism of each, as specific phenotypes can be used to more readily assign gene function compared to pleiotropic genes [51]. One example is the cluster ABZR88_RS14505-ABZR88_RS14520 (ribulokinase, L-ribulose-5-phosphate 4-epimerase and L-arabinose isomerase), which is important for fitness on L-arabinose. These three genes correspond to the arabinose operon first described in *E. coli* [62]. Within this cluster, a predicted GH27 enzyme (ABZR88_RS14515) had no clear impact on fitness, despite more than 50% identity to β-L-arabinofuranosidases. One possible explanation may be the presence of a possibly redundant gene in the YX-36 genome (i.e. ABZR88_RS12975, 31% homology), which could mask the phenotype due to compensation. Other cases are ABZR88_RS03755 and ABZR88_RS13180-ABZR88_RS13190, involved in fructose metabolism and D-cellobiose metabolism, respectively.

Of the four polysaccharides we profiled, we could link two to at least one PUL, because multiple genes in the PUL were important for fitness on that substrate. First, we found that PUL32 was mildly important for growth on alginate, with five genes (ABZR88_RS13305-AZBR88_RS13325) with significant phenotypes. In contrast to the single PUL we identified for alginate, we found that four different PULs were important for growth on polygalacturonic acid (PUL3, PU14, PUL19, PUL20), although not all predicted genes within each PUL were required for fitness. In PUL3, the SusCD pair was not important, but other genes including ABZR88_RS02125 (pectinesterase family protein) had mild but significant phenotypes. In PUL14, only the *susCD* genes (ABZR88_RS06240-ABZR88_RS06245) were important for growth on polygalacturonic acid, while the other genes were dispensable. While PUL19 is predicted to encode two separate SusCD pairs, only the ABZR88_RS08375-ABZR88_RS08380 system was important for fitness. Lastly, in PUL20, three of the five predicted genes including the SusCD homologs (ABZR88_RS08490-ABZR88_RS08500) were required for optimal growth on the polysaccharide. Our results demonstrate that in certain instances, components of multiple PULs can contribute to the catabolism of a complex polysaccharide.

Although our main focus was genes with a negative fitness, we also analyzed detrimental genes. We found that a cluster (ABZR88_RS07190-ABZR88_RS07205 within PUL16, Table S4) coding for two pairs of GH2 and GH3 enzymes is detrimental (fitness ≥1) for metabolism of D-melibiose, a disaccharide present in plants. While melibiose breakdown requires an ⍺-galactosidase, these four enzymes are β-glycosidases, which close homologs rather suggest the degradation of polysaccharides made of β-galactose, β-glucose, and β-glucuronic acid, such as in soil rhizobial exopolysaccharides [63]. This phenotype is exclusive to transposon mutations inserting on the positive strand of these genes, and the probable overexpression of the downstream gene ABZR88_RS07210 (coding for an alpha-galactosidase). In support of this, ABZR88_RS07210 is mildly important for melibiose utilization, and thus overexpression of this gene would provide a fitness benefit on this substrate. This finding very closely mirrors what was previously observed in *B. thetaiotaomicron* in mice, where transposon mutants driving overexpression of a gene important for melibiose utilization have a large competitive advantage [64]. Another example is a cluster (ABZR88_RS04740-ABZR88_RS04750) that is detrimental for metabolism of D-xylose, D-cellobiose, D-xylose, D-arabinose, polygalacturonic acid, and gliding. One of these genes belongs to family GT2, questioning the possible importance of the glycosylation of gliding proteins, similarly to motility by flagella in Gram-negative [65].

In total, we identified genes involved in carbon metabolism (|fitness ≤1|) in 15 out of the 45 different PULs in strain YX-36 (Table S2, Table S4). In the future, similar experiments with additional complex glycans, including some of the main polysaccharides secreted from plant roots (e.g. hemicelluloses such as xyloglucan and heteroxylans) [66, 67] could provide insightful information to understand gene function and reannotate hypothetical genes in strain YX-36.

#### Gliding motility

T9SS is tightly linked to gliding motility through the involvement of the T9SS in adhesin export and possibly through the use of a shared motor complex [5, 56, 68]. The ability to glide on surfaces without the aid of external appendages such as flagella, is widespread among Bacteroidota and it allows an active search for nutrients as well as rapid secretion and release of CAZymes into the environment [5, 68].

The central element of the T9SS is SprA, which is the largest single polypeptide transmembrane β-barrel identified to date [69]. Some other components of the T9SS are GldKLMN and PorQUVZ (Fig. 3A). The exact function of many of the T9SS components are unclear. In *M. yixingensis* YX-36, there are more than 100 genes annotated as related to gliding and T9SS. The gliding (GldBDJ, SprT) and T9SS (GldKLMN, SprAE) mandatory components are present in this strain, as we assessed with the T9GPred [70] tool (Fig. 3B), meaning that YX-36 has T9SS and is able to glide.

We confirmed that the predicted mandatory gliding genes GldBD, SprT (ABZR88_RS20050, ABZR88_RS15825, ABZR88_RS04210 (Fig. 3B) were important (fitness ≤1) for gliding motility in strain YX-36 (Table S2). Gene GldJ (ABZR88_RS01485) was not identified as involved in gliding in our screening, but its homolog ABZR88_RS04130 was. Other expected genes that we found are ABZR88_RS01480 (PorV), ABZR88_RS04135 (GldJ homolog), ABZR88_RS04135 (GldL), ABZR88_RS04140 (GldM), ABZR88_RS04145 (GldN), ABZR88_RS16235 (GldG), and ABZR88_RS18315 (cell surface protein SprA) (Table S2). Many of the above genes have high positive cofitness (i.e. correlation between their fitness patterns, and high probability of shared functions), and their homologues have previously been related to gliding motility in Bacteroidota [71]. We also identified novel genes related to gliding motility: ABZR88_RS02055 (signal peptidase I), ABZR88_RS20165 (ribosome silencing factor), and ABZR88_RS00640 (serine hydroxymethyltransferase), the latter being important for growth in multiple other conditions as well, including plant colonization (Table S2).

#### Stress tolerance

*Mucilaginibacter* species are resistant to diverse stresses [25, 50, 72]. One possible explanation of this tolerance is the abundant exopolysaccharides that they synthesize [73]. We assayed *Mucilaginibacter*_YX36_ML5 in different stress conditions: temperature (i.e. 15°C, 21°C, 30°C, 37°C), metal salts (i.e. NaCl, CuCl_2_, KF, K_2_HPO_4_), antibiotics (i.e. kanamycin), and plant-related molecules (i.e. 6-methoxy-2(3H)-benzoxazolone or MBOA, quercetin, apigenin, esculin).

From these data, we found that ABZR88_RS11280 (TonB-dependent receptor) was important (fitness ≤1) for tolerance to quercetin (and mildly important for gliding motility), but not the adjacent TonB-dependent receptor (ABZR88_RS11275). We also found that ABZR88_RS11640 (sodium-translocating pyrophosphatase), ABZR88_RS22105 (voltage-gated chloride channel family protein), and ABZR88_RS14150 (magnesium transporter) were needed for growth in presence of KF. Homologs of both of these genes were also required for fluoride tolerance in *Pedobacter* sp.GW460-11-11-14-LB5 [51]. We also identified phenotypes for several genes coding for RND (resistance-nodulation-cell division) family transporters [74], such as the efflux RND transporter periplasmic adaptor and permease pair ABZR88_RS03435-ABZR88_RS03440, which was detrimental for stress tolerance to CuCl_2_ (Table S2).

Interestingly, we identified that several components of the Bacteroidota-specific T9SS, namely ABZR88_RS01480 (outer membrane channel protein PorV), ABZR88_RS18315 (SprA), ABZR88_RS04140 (gliding motility protein GldM), ABZR88_RS04145 (GldN), ABZR88_RS04210 (GldB), ABZR88_RS20050 (GldD), and ABZR88_RS16235 (GldG) are needed for tolerance to the plant-associated stressors MBOA, quercetin, apigenin, and high K_2_HPO_4_ (Table S2). Many of these genes have high positive cofitness.

As other Bacteroidota, members of the *Mucilaginibacter* genus are also very resistant to different antibiotics [75]. Strain YX-36 is resistant to kanamycin, among others. Interestingly, it was recently described that strain *Mucilaginibacter* 21p has cross-resistance mechanisms that confer resistance to both antibiotic and metal salts [50]. In agreement with this, we observed that genes ABZR88_RS12790 (glycosyltransferase), ABZR88_RS12785 (flippase), ABZR88_RS17545 (glycosyltransferase GT2 family), ABZR88_RS09925 (ABC transporter ATP-binding protein), ABZR88_RS09930 (ABC transporter permease), ABZR88_RS12795 (UDP-galactopyranose mutase), and ABZR88_RS17535 (oligosaccharide flippase family protein) are all important for tolerance to the salts KF and NaCl and the antibiotic kanamycin, but not for other stressors such as CuCl_2_, apigenin, quercetin or MBOA (Table S2). These genes are probably involved in the import of components for the production and externalization of a polysaccharide biofilm key to bacterial protection and nutrient storage.

### Identification of plant colonization genes in strain YX-36

**Figure 4:**
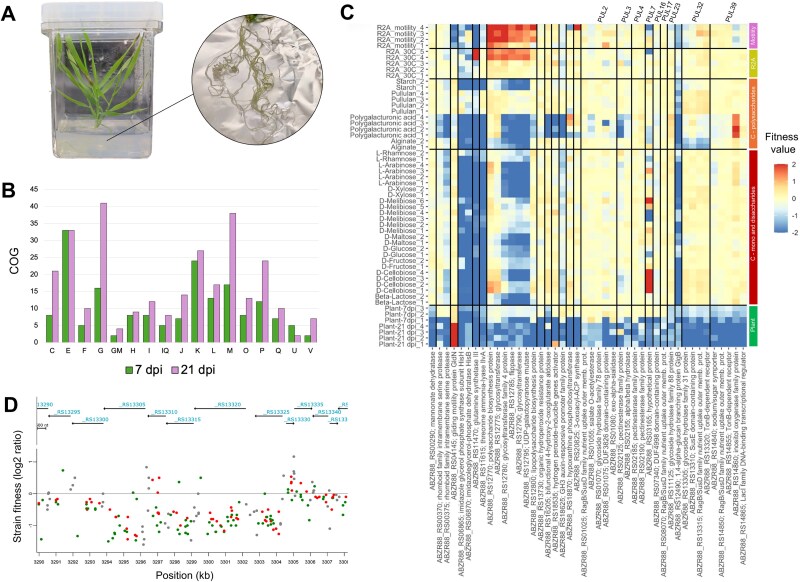
(A) Overview of *Brachypodium distachyon* Bd21-3 plants, and of the plant rhizosphere after harvesting. (B) Clusters of orthologous groups (COG) enrichment analysis of bacterial genes associated with plant samples collected at 7 and 21 days post-inoculation (dpi). For illustration purposes, only the most different categories are shown. (C) Heatmap of fitness values for a selection of plant rhizosphere colonization genes across different plant and *in vitro* tests. Gene fitness values are bounded from −2 to 2. For visualization purposes, temperature and stress assays are not shown. (D) Strain fitness for selected genes (from ABZR88_RS13290 to ABZR88_RS13345) averaged across three plant assays. Strains in the central 10%–90% of a gene are color coded by the orientation of the transposon insertion (green represents forward strand, and red reverse strand). Strains with intergenic insertions or insertions near the edge of a gene are not used in the gene fitness calculation and are shown as grey dots.

*Mucilaginibacter* species have been isolated from the root and rhizosphere of different plants, such as *Brachypodium*, switchgrass, and rice [41, 43, 48, 76]. Some strains have been found to be positively correlated with crop biomass and yield [77]. Others are increased in abundance and proportion within a community when the amount of exuded compounds increases [78]. Recently, we described a *Mucilaginibacter* strain that could efficiently colonize the rhizosphere of *B. distachyon* plants in competition with other bacterial species [42]. Although numerous studies focus on the identification of plant colonization fitness genes in bacteria belonging to the phylum Pseudomonadota [79], few or no data are available on plant-related Bacteroidota. With the aim to bridge this gap and identify plant colonization strategies in environmental Bacteroidota, we evaluated the fitness of *Mucilaginibacter*_YX36_ML5 in the rhizosphere of *B. distachyon* plants at 7 and 21 dpi (Fig. 4A). This was made to consider the dynamism of the colonization process [64]. On a global scale, our data indicate that a greater number of genes are specifically implicated in plant colonization at 21 dpi compared to 7 dpi (Table S2), potentially reflecting a more stringent adaptive response to the rhizosphere environment or altered nutrient availability. Genes related to COG (Clusters of Orthologous Groups) [80] categories C (energy production and conversion), G (carbohydrate transport and metabolism), J (translation, ribosomal structure and biogenesis, M (cell wall/membrane/envelope biogenesis), and P (inorganic ion transport and metabolism) (Fig. 4B, Table S2) are differentially enriched over time (i.e. more important for colonization of plants 21 dpi than for plants 7 dpi), possibly reflecting temporal shifts in bacterial adaptation and persistence within the plant environment. Nonetheless, additional investigations incorporating more temporally distant sampling points are necessary to substantiate these observations.

Several of the genes that we found to be involved (|fitness| ≥ 1) in plant colonization by strain YX-36 are related to amino acid synthesis. Examples are ABZR88_RS06865-ABZR88_RS06870 (imidazole glycerol phosphate synthase and dehydratase, HisH and HisB), ABZR88_RS11470 (glutamine synthetase), and ABZR88_RS11615 (threonine ammonia-lyase IlvA) (Fig. 4C, Table S2). Orthologues of these genes have previously been associated with plant colonization in phylogenetically distant species [79, 81, 82], which shows that amino acid synthesis is required by different bacteria for efficient plant colonization [79]. Genes within other functional categories that we find involved (|fitness| ≥ 1) in rhizosphere survival are ABZR88_RS00290 (mannonate dehydratase), ABZR88_RS16205 (bifunctional 4-hydroxy-2-oxoglutarate aldolase), ABZR88_RS12770-ABZR88_RS12800 (lipopolysaccharide biosynthesis proteins), and ABZR88_RS04145 (gliding protein GldN). They correspond to phenotypes commonly found in plant-associated bacteria, such as biofilm formation and motility [83, 84]. Other examples are ABZR88_RS18870 (hypoxanthine phosphoribosyltransferase), ABZR88_RS18625 (GH3 auxin-responsive promoter family protein), ABZR88_RS20825 (3-oxoacyl-ACP synthase), involved in traits such as purine recycling, plant hormone signaling, and quorum sensing/fatty acid synthesis, which are all traits related to successful rhizosphere colonization [85, 86]. Finally, we found that both genes ABZR88_RS00370-ABZR88_RS00375, coding for rhomboid family intramembrane serine proteases, are important for root colonization (Fig. 4C). This family of genes is involved in cleaving orphan proteins to prevent aggregation in the cell and has been described in *E. coli* as needed for mice colonization, although through unknown mechanisms [87].

Interestingly, our findings reveal that largely phylum-specific genes (e.g. PULs, CAZymes) are needed for fitness in the plant niche, allowing Bacteroidota strains privileged access to nutrients in the rhizosphere that remain inaccessible to other phyla like Pseudomonadota. Examples are the genes ABZR88_RS02185-ABZR88_RS02190 (pectinesterase family proteins, CAZyme family CE8, in PUL4), ABZR88_RS11125 (glycoside hydrolase, CAZyme family GH88, in PUL23), and ABZR88_RS12490 (⍺-1,4-glucan branching protein GlgB, displaying families CBM48 and GH13 domains, and related to ⍺-glucan metabolism), the later showing negative fitness in the presence of multiple carbon sources including D-xylose, D-fructose, and L-arabinose. Another example of a plant-associated phylum-specific cluster is ABZR88_RS13305-ABZR88_RS13325, coding for a glycoside hydrolase GH31 family protein, a SusE domain-containing protein, a RagB/SusD family nutrient uptake outer membrane protein, a TonB-dependent receptor and a DUF6377 domain-containing protein (Fig. 4C, Table S4). Interestingly, these genes, all belonging to PUL32, are also important for growth on alginate (Fig. 4C and D). We do not expect alginate (produced by seaweed) to be present in our experimental system (either from the plant or in the agar), and it is more likely that PUL32 is important for growth on additional polysaccharides. Several genes within PUL2, PUL4, PUL7, PUL16, and PUL17 also were needed for colonization of the plant niche (Fig. 4C, Tables S2 and S4). Finally, we also identified a group of genes belonging to PUL39 that were mildly but significantly important for plant colonization: ABZR88_RS14840 (sodium/sugar symporter), ABZR88_RS14850 (RagB/SusD family nutrient uptake outer membrane protein), ABZR88_RS14855 (TonB-dependent receptor), ABZR88_RS14860 (inositol oxygenase family protein), and ABZR88_RS14865 (LacI family DNA-binding transcriptional regulator) (Fig. 4C, Table S2). The specific substrate(s) recognized by PUL39 are not clear, as we did not detect a phenotype for these genes in our *in vitro* dataset, and close homologs of these proteins have not yet been characterized.

Our results demonstrate that efficient plant colonization in YX-36 requires the activity of multiple PULs, providing evidence that proteins characteristic of Bacteroidota are important for this process, likely mediated via the degradation of polysaccharides and their breakdown products. Martin *et al.* [10] demonstrated that plant Bacteroidota lack high-affinity transporters for uptake of simpler carbon compounds like monosaccharides, but contain multiple *susCD*-like complexes, presumably for utilization of polysaccharides in the rhizosphere. Their results are in agreement with our findings and the conclusion that genes relating to CAZymes and PUL are important for plant colonization. Future experiments could focus on deeper characterization of the identified PULs important for *Brachypodium* colonization, as well as including more diverse plant-secreted polysaccharides [66, 67] and plant species to determine if these colonization genes are specific to certain hosts.

## Conclusion

Bacteroidota species are trending in microbiology research due to their exceptional adaptive capacity to proliferate in diverse environments [4, 88], yet the genetic determinants underlying their various lifestyles are not yet clear. In spite of the interest raised in the past years in the environmental members of this phylum, it remains a challenge to study the pathways and mechanisms related to fitness in plant hosts.

In this study, we succeeded in generating a genome-wide mutant library in *M. yixingensis* YX-36. Notably, our results highlight that phylum-specific genes (e.g. PULs) are important for plant colonization and may give Bacteroidota unique access to nutrients or niches unavailable to other phyla. We present here strong candidates for further functional characterization through controlled *in vitro* and in vivo experiments and present an exciting avenue for further research. These future investigations will be strengthened with the development of genetic systems for targeted gene deletion or disruption, thus enabling follow-up studies with individual mutant strains. Deciphering the poorly explored functional potential of plant-associated Bacteroidota will contribute to the development of novel environmental applications of this understudied bacterial phylum. We hope that our mutant library will be a valuable resource for the community doing research in the poorly characterized genus *Mucilaginibacter*, that our workflow will be adapted to additional environmental members of this phylum, and that our results will draw more attention to environmental Bacteroidota, especially those living in close association with plants and food crops.

## Supplementary Material

Supplementary_material_ycag186

## Data Availability

The raw data generated during and analyzed during the current study is available at https://doi.org/10.6084/m9.figshare.30731249.v1.
