# Supplementary material for: Unraveling fitness landscapes in plant-associated Bacteroidota
Source: ISME Commun. 2026 Jul 1;6(1):ycag186. doi: 10.1093/ismeco/ycag186 (PMC13440311; doi:10.1093/ismeco/ycag186)
Supplement: Supplementary file 1 [file supplementary_material_ycag186.zip › 3_Supplementary Information_June2026.docx]

**Supplementary Information**

**Unraveling Fitness Landscapes in Plant-Associated Bacteroidota**

Marta Torres^1,#^, Vincent Lombard^2,3^, Nicolas Terrapon^2,3^, Adam M. Deutschbauer^1,4,#^

*^1^Environmental Genomics and Systems Biology Division, Lawrence Berkeley National Laboratory, Berkeley, CA 94720, USA.*

*^2^Aix Marseille University, CNRS, UMR7257, AFMB, Marseille, 13288, France*

*^3^INRAE, USC1408 AFMB, Marseille, 13288, France*

*^4^Department of Plant and Microbial Biology, University of California, Berkeley, CA 94720, USA.*

^#^*Corresponding authors:* [*mtorresbejar@gmail.com*](mailto:mtorresbejar@gmail.com)*,* [*amdeutschbauer@lbl.gov*](mailto:amdeutschbauer@lbl.gov)

*Marta Torres/Adam Deutschbauer, Environmental Genomics and Systems Biology Division, Lawrence Berkeley National Laboratory, 1 Cyclotron Road*

*Berkeley, CA 94720, USA.*

ORCID identifiers

*Marta Torres 0000-000-0609-9123*

*Nicolas Terrapon 0000-0002-3693-6017*

*Vincent Lombard 0000-0002-9684-7067*

*Adam M. Deutschbauer 0000-0003-2728-7622*

**Running title**

Genetic Basis of Fitness in *Mucilaginibacter*

**Figure S1.** Overview of the methodology and the steps followed in this study to construct a mutant library in *Mucilaginibacter yixingensis* YX-36.

**Figure S2**. Predicted polysaccharide utilization loci (PULs) in *Mucilaginibacter yixingensis* YX-36 as determined by the PUL database (PULDB).

**Figure S3.** Total number of CAZymes among the 41 *Mucilaginibacter* strains in the CAZyme database. GH, glycosyl hydrolase; GT, glycosyl transferase; PL, polysaccharide lyase; CE, carbohydrate esterase; CBM, carbohydrate binding module.

**Figure S4**. Distribution of CAZymes among the 41 *Mucilaginibacter* strains in the CAZyme database. GH, glycosyl hydrolase; GT, glycosyl transferase; PL, polysaccharide lyase; CE, carbohydrate esterase; CBM, carbohydrate binding module.

**Table S1**. Summary of fitness assays conducted with *Mucilaginibacter*_YX36_ML5.

**Table S2**. Fitness data of *Mucilaginibacter yixingensis* YX36. Fitness values and t scores are shown. Assays publicly available at the Fitness Browser website (https://fit.genomics.lbl.gov) are noted.

**Table S3**. *Mucilaginibacter*_YX36_ML5 gene information, including scaffold, begin, end, strand, GC content of the gene's sequence and nTA (number of TA dinucleotides in the gene's sequence; the *mariner* transposase usually inserts at TA).

**Table S4**. Predicted PULs and CAZymes in the genome of *Mucilaginibacter yixingensis* YX-36.

**Study funding**

This material by m-CAFEs Microbial Community Analysis & Functional Evaluation in Soils (<https://mcafes.lbl.gov>), a Science Focus Area led by Lawrence Berkeley National Laboratory, is based upon work supported by the U.S. Department of Energy, Office of Science, Office of Biological & Environmental Research under contract number DE-AC02-05CH11231. Genome sequencing of strain YX-36 was funded by ENIGMA Ecosystems & Networks Integrated with Genes and Molecular Assemblies (<https://enigma.lbl.gov/>), supported by the Office of Science, Office of Biological and Environmental Research of the U.S. Department of Energy, under contract DE-AC02-05CH11231.nded by conducted by ENIGMA was supported by the Office of Science, Office of Biological and Environmental Research of the U.S. Department of Energy, under contract DE-AC02-05CH11231.
